# Visualization, Dynamicity, and Collaborative Networking of Scientific Production on Visible Light and Skin Aging: A Scientometric Analysis

**DOI:** 10.1155/2024/5589020

**Published:** 2024-10-03

**Authors:** Fran Espinoza-Carhuancho, Carlos Quispe-Vicuña, Cesar Mauricio-Vilchez, Diego Galarza-Valencia, Julia Medina, Josmel Pacheco-Mendoza, Frank Mayta-Tovalino

**Affiliations:** ^1^ Bibliometrics Evidence Evaluation and Systematic Reviews Group (BEERS) Human Medicine Career Universidad Científica del Sur, Lima, Peru; ^2^ Academic, Department Clinical and Health Effectiveness Network (REDECS), Lima, Peru; ^3^ Academic Department Faculty of Medical Technology Universidad Nacional Federico Villarreal, Lima, Peru; ^4^ Research Innovation and Entrepreneurship Unit Universidad Nacional Federico Villarreal, Lima, Peru; ^5^ Systematic Reviews and Meta-analysis Clinical Practice Guidelines and Health Technology Assessment Unit Vicerrectorado de Investigación Universidad San Ignacio de Loyola, Lima, Peru

## Abstract

**Purpose:** The purpose of this is to examine the visualization, dynamicity, and collaborative networking of scientific production on visible light (VL) and skin aging through scientometric analysis.

**Materials and Methods:** This research consisted of a cross-sectional and descriptive design with a scientometric approach that examined the publication trends and collaborative patterns among authors and institutions from 2018 to 2023. A comprehensive search strategy was also employed by using specific keywords related to VL and skin aging. In this case, several indicators were employed, including scholarly output, view count, field-weighted citation impact (FWCI), and citation count. The analyses were performed by using SciVal software and R Studio version 4.3.2.

**Results:** A total of 180 sources were identified, with 280 documents generated, indicating an annual growth rate of 6.72%. The documents, averaging 3.25 years in age, received an average of 12.14 citations, revealing their impact. Additionally, collaborations were evident, with a ratio of 5.6 coauthors per paper and 25.71% consisting of international collaborations. In terms of institutions, there were notable disparities in scholarly activities and impact metrics, highlighting the diversity of the research landscape. Meanwhile, journals, such as *Photodermatology, Photoimmunology and Photomedicine*, revealed a substantial impact (FWCI 2.05). Overall, the impact of the journals showed a general upward trend, reflecting dynamicity and variability over time.

**Conclusion:** An annual growth rate of 6.72% was found, with 180 sources and 280 papers on VL and skin aging. Moreover, international collaborations, the positive impact in leading journals, and the distribution patterns identified through scientometric laws underscored the vitality and complexity of the field. These results offer valuable insights into guiding future research in this multidisciplinary field.

## 1. Introduction

Visible light (VL) refers to the spectrum of electromagnetic radiation perceived by the human eye that normally encompasses a wavelength range of 400–700 nm and allows the perception of colors [1]. While approximately half of VL is mainly constituted by sunlight, the remainder is constituted by artificial sources such as flashlights, lasers, and fluorescent lights [2]. However, electronic devices, such as smartphones, laptops, and computers, are becoming frequent sources of radiation. In this regard, the skin is affected by exposure to the generated VL, since it is the body's first barrier. Meanwhile, exposure to VL favors the development of pigmentary disorders in the skin [3], such as vitiligo or melasma, and the development of neoplastic processes, such as melanoma. In recent years, there have been 325,000 new cases of melanoma, with the number of cases expected to rise [4].

Although it was previously believed that VL did not significantly affect human biology (other than skin pigmentation), it has recently been reported that it is related to the activation of chromophores, oxidative stress, circadian rhythm, and photoaging [5, 6]. Regarding the latter, skin photoaging is a complex process that involves degenerative (hyperpigmentation and decreased elasticity) and biochemical changes (dysfunction of the nucleus, mitochondria, DNA damage, and generation of free radicals) [7]. Despite these aspects, the previous studies have mainly focused on the effects of ultraviolet (UV) radiation on disease generation, ignoring its effects on the skin, especially at the biological level [7]. This clarifies the gap in knowledge that exists regarding the effects of UV on photoaging. To address this gap, an analysis of the productivity in the medical literature to date should be performed by using a bibliometric approach. This allows a quantitative analysis of a given topic/area of study and assesses the predominant trends in research [8]. However, although previous bibliometric studies have evaluated VL productivity, they only focused on its therapeutic application by means of light emitting diode (LED) sources [9].

Therefore, the purpose of this study is to analyze the visualization, dynamicity, and collaborative networking of scientific production on VL and skin aging through scientometric analysis. The scientific rationale for this study is that skin aging, a complex phenomenon with a significant impact on health and the quality of life, is intrinsically linked to VL exposure. It is hoped that the findings will provide a more current overview of the productivity of VL in cutaneous aging in order to improve health policies that favor the management of patients with cutaneous alterations due to VL.

## 2. Materials and Methods

### 2.1. Study Design

This research consists of a cross-sectional and descriptive design with a scientometric approach that examines the publication trends and collaborative patterns among authors and institutions from 2018 to 2023.

### 2.2. Data Selection

For the data selection, this study performed a search of the Scopus database on February 2, 2024, by using the formula in Table 1.

### 2.3. Inclusion Criteria

• Original research papers published in peer-reviewed journals.
• Systematic reviews, meta-analyses, and narrative reviews that provide a comprehensive overview of the literature on VL and skin aging.
• Published between January 1, 2018, and December 31, 2023.
• The study must be published in English.

### 2.4. Exclusion Criteria

• Studies with methodological flaws or those that lack adequate controls are excluded.
• Studies published in languages other than English are excluded.
• Studies published in other databases.

### 2.5. Data Extraction

The information retrieved included 205 articles, 51 reviews, 13 book chapters, 7 conference papers, 3 short surveys, and 1 conference review, providing a solid basis for a detailed scientometric analysis. The data extracted from the Scopus database was subjected to a dual analysis by using SciVal and Bibliometrix software. This involved assessing publication trends, geographic distribution, institutional contributions, and author productivity. SciVal's advanced features, such as citation impact analysis and field-weighted citation impact (FWCI), were used to assess the relative impact of publications within their respective fields. SciVal's visualization capabilities were also applied to create charts and graphs of the research landscape and its evolution over time. Meanwhile, Bibliometrix, an R package for the comprehensive analysis of scientific mapping, was used to conduct a more in-depth bibliometric analysis. Specifically, Bibliometrix's network analysis tools were used to identify collaborative patterns among authors and institutions, revealing the research collaborations in the field of VL and skin aging. By using both tools, it not only provided a comprehensive understanding of the bibliometric landscape of VL and skin aging research, but it also shed light on publication trends, collaboration patterns, thematic foci, and the overall impact of research in this field.

### 2.6. Scientometric Analysis

In this study, the indicators included (1) scholarly output, which quantifies the number of documents published by each country/region; (2) view count, which provides a measure of the scope and visibility of academic production; (3) FWCI, a standardized metric that evaluates the impact of the documents by considering the relevance of the field of study; and (4) citation count, which quantifies the total number of citations received by the documents. All of the analyses were performed by using SciVal software (2024 Elsevier B.V.) and R studio version 4.3.2 (2023-10-31 ucrt).

## 3. Results

The scientometric analysis showed that between 2018 and 2023, a total of 180 sources (including journals and books) were identified, with 280 documents generated. In this regard, the annual growth rate was 6.72%, reflecting significant dynamism in academic production. The average age of the documents was 3.25 years, with an average of 12.14 citations, indicating considerable impact and relevance in the scientific community. Additionally, collaborations among the authors were evident, with a ratio of 5.6 coauthors per paper. Among these collaborations, 25.71% were international, underscoring the global nature of the research. In terms of paper types, 205 articles, 13 book chapters, 7 conference papers, 1 conference review, and 51 reviews were found, in addition to 3 short surveys. These results provide a comprehensive overview of the diversity and richness of scientific production in the sample period (Table 2).

When examining the institutions, the results revealed notable disparities in scholarly activities and impact metrics. The Centre National de la Recherche Scientifique (representing the government sector in France) was a prominent contributor with 15 scholarly outputs, accumulating 399 views and a FWCI of 1.64, indicating a significant influence on the academic landscape. Conversely, the Russian Academy of Sciences (also a governmental institution) had a relatively low scholarly output of 6, with 153 views and a remarkably low FWCI of 0.69. Moreover, the University of Alcalá in Spain (an academic institution) exhibited a remarkable FWCI of 3.19, underscoring the substantial impact of its 7 scholarly outputs. These bibliometric insights illuminate the diverse research landscapes across institutions, sectors, and nations, providing a nuanced understanding of their scholarly contributions and influences (Table 3).

When evaluating academic output by scientific journal, the *International Journal of Molecular Sciences* and the *Journal of Cosmetic Dermatology* exhibited comparable academic outputs of 8, but differed in their citation impacts, i.e., 1.18 and 1.79, respectively. Notably, *Photodermatology, Photoimmunology and Photomedicine* stood out with a solid FWCI of 2.05, along with 7 scholarly productions and 201 views, indicating its substantial impact in the field. In contrast, *Scientific Reports* had a lower FWCI of 0.5, along with 6 scholarly productions, suggesting a mixed impact, despite a relatively high citation count of 107. Other journals, such as *Biomedical Optics Express* and *ACS Applied Materials & Interfaces* found a balance between production volume and impact, demonstrating a moderate FWCI of 0.79 and 1.46, respectively. These bibliometric assessments not only provide a nuanced understanding of the academic landscape, but they also illustrate the varying degrees of impact and participation associated with different sources in the Scopus database, especially in the molecular sciences, dermatology, and related disciplines (Table 4).

**Figure 1:**
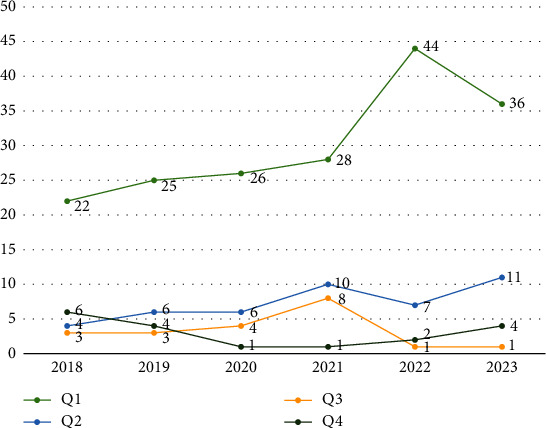
Impact of publications according to quartile (CiteScore).

The impact of journals also showed a general upward trend from 2018 to 2023. In the first quartile (Q1), a significant increase was observed from 22 in 2018 to 44 in 2022, although it decreased slightly to 36 in 2023. The second quartile (Q2) and fourth quartile (Q4) showed minor fluctuations over the years. Surprisingly, the third quartile (Q3) experienced a peak in 2021, with a value of 8, but sharply declined to 1 in the following 2 years. In total, the CiteScore increased from 35 in 2018 to 54 in 2022 and then slightly decreased to 52 in 2023. This data reflects the dynamics and variability of the impact of journals over time (Figure 1).

**Figure 2:**
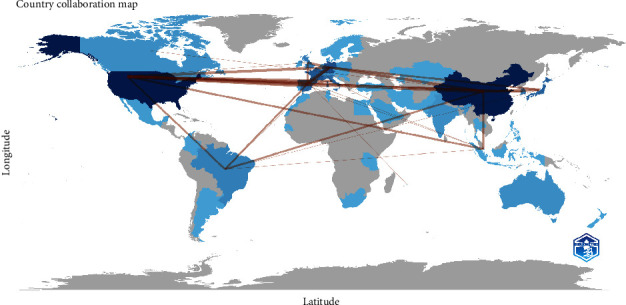
Country collaboration map.

As for the mapping of collaborations between countries, it revealed interesting patterns in academic interactions at the global level. Several partnerships stood out, such as the collaborations between Argentina and Austria, Australia and Hungary, and Australia and Mauritius, all with a one-time collaboration. Brazil demonstrated a wide collaborative network with various countries, such as Argentina, Austria, Ireland, Mexico, Portugal, Senegal, and Singapore, indicating its active participation in the international scientific cooperation. Meanwhile, China emerged as a central player, collaborating with the United States, as the most frequent partner (6 collaborations), followed by Germany, Japan, and Singapore (4, 4, and 3 collaborations, respectively). This diversity in collaborations underscores China's significant influence on the global scientific scene. As for the United States, it was closely associated with countries such as Spain (6 collaborations), Italy (4 collaborations), Germany (4 collaborations), and Japan (3 collaborations). These relationships highlight the importance of the international cooperation in scientific research (Figure 2).

**Figure 3:**
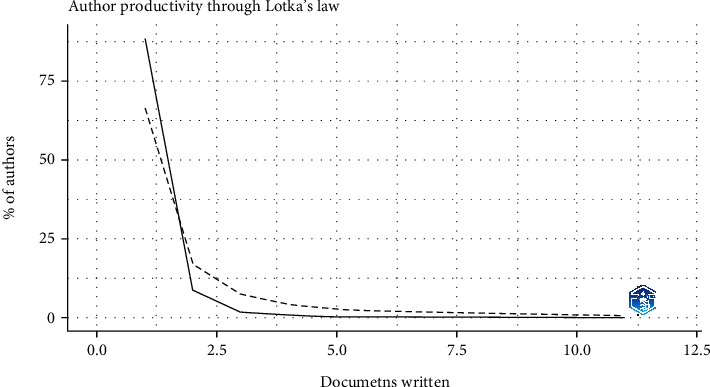
Author productivity.

Lotka's law revealed a distribution pattern that confirms the trend in the scientific literature. In this case, 1192 papers were written by a single author. However, as the number of authors increases, the frequency of papers significantly decreases, indicating an inverse relationship. Conversely, the second largest group consisted of documents written by two authors, with 115 cases. These results corroborate that most of the contributions were from a small group of authors and that collaborations between a larger number of authors were less common (Figure 3).

**Figure 4:**
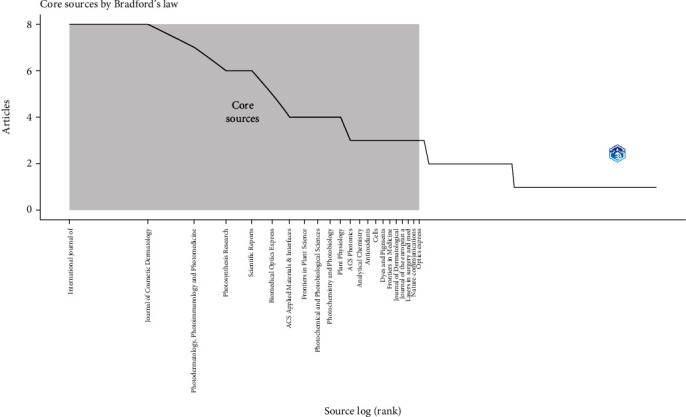
Core sources.

Finally, Bradford's law revealed that Zone 1, which comprises the top five journals, led by the *International Journal of Molecular Sciences* and the *Journal of Cosmetic Dermatology*, accounted for 25% of the total cumulative frequency. This indicates a significant concentration of activity in a small number of leading journals. In contrast, Zone 2 included a larger group of journals, where positions 6 to 23 contributed the next 25% of the total cumulative frequency. This range shows a more even distribution, with several journals similarly contributing to the total output. Finally, Zone 3 encompassed a wide variety of journals, from position 24 to the last position (Figure 4).

## 4. Discussion

Between 2018 and 2023, this study found 280 papers (mainly articles and reviews), with an annual growth rate of 6.72% and an average citation of more than 12 citations per article. The papers were found to have (on average) more than 5 authors per article and were mostly published in high quartile journals. In addition, collaborations were mainly international, with a higher impact from China and the United States.

Overall, the University of Alcalá in Spain was the institution with the highest impact, despite having only 7 publications. Its last publication with the highest number of citations [10] was the study by González et al. [11] which provided expert recommendations on the efficacy of sunscreens and the beneficial role of nonfiltering ingredients such as biological/nonbiological extracts. This objective is relevant in the context of skin aging, since it is known that ultraviolet A (UVA) rays from the sun not only favor the development of up to 90% of skin cancers [10] but also favor the expression of matrix metalloproteinase, which degrades elastin and collagen in epithelial cells and promotes skin aging [12]. In this sense, the fact that sunscreens specifically protect against UVA rays provides a protective function against skin aging.

It was also evident that the *Journal of Cosmetic Dermatology* had the highest productivity and impact, despite having only 8 publications. In this journal, the publication with the highest number of citations (121) was the review by McDaniel, Farris, and Valacchi [13], which evaluated the atmospheric factors that condition skin aging such as sun exposure and air pollution. In this regard, environmental exposure causes oxidative damage to the cellular components of the skin such as proteins, lipids, and nucleic acids. Another journal that also accounted for a quarter of the productivity on the topic of VL and skin aging was the *International Journal of Molecular Sciences.* This journal was reported in several bibliometric studies to be the most productive in terms of cellular processes and their association with diseases [14, 15]. This correlates with the fact that its most cited publication (67 citations) with respect to the subject of the present study was the review by Parrado et al. [16], which focused on an extract of *Polypodium leucotomos* (Fernblock) and evaluated its molecular mechanisms and pleiotropic effects on light-related skin conditions, photoaging, and skin cancers.

There were mainly international collaborations in high quartile journals, demonstrating a global interest in the impact of VL on skin aging. The countries with the largest collaborative network included the United States, China, and Brazil, correlating with the fact that these countries have recently experienced an increase in health-damaging UV doses [17–19].

Overall, these results suggest that despite some evidence demonstrating the impact of VL on skin aging and overall health, future research should evaluate this effect either through in vivo studies to determine the mechanism and spectrum of action of photoaging in humans or through optimal photoprotection studies to evaluate VL wavelengths [20]. It is hoped that the results of our study will serve as a basis not only for future studies but also for strengthening the prevention of pigmentary diseases by physicians and increasing public awareness of the symptoms of skin aging.

The scientific justification for this study lies in its bibliometric nature, which seeks to analyze and quantify scientific production in the field of VL and skin aging. Unlike a clinical study, which focuses on establishing causal relationships between variables, a bibliometric analysis focuses on evaluating research trends, collaboration patterns, and the impact of publications in a specific area of study. Therefore, it is not necessary to demonstrate a causal relationship between LV and skin aging. Instead, the aim is to provide an overview of scientific productivity in this field, identify major areas of focus and collaboration, and highlight gaps in existing research. This could help guide future research and health policy and make a valuable contribution to the multidisciplinary field of cutaneous aging and VL.

This study includes some limitations that should be noted. First, the search was limited to the Scopus database, which may have excluded relevant articles from other gray literature databases. Second, since the search was conducted between 2017 and 2024, publications prior to this date were not included. Third, the classification of the documents (articles, reviews, letters, book chapters, etc.) may not fully reflect their contents or methodologies. However, this study includes several strengths. First, the Scopus database covers many journals that undergo a strict peer-review process to ensure their high quality, compared with other databases [21]. Second, this study provides an updated view of the research by taking 2017–2024 as the sample period. Third, by classifying the studies according to the type of publication, it allows for a more complete and diverse view of the field of study. Most importantly, since this is the first study to assess productivity on VL and skin aging, it is hoped that it will provide a crucial overview [22, 23] of the knowledge gap and serve as a basis for future research.

## 5. Conclusion

This scientometric study provided a comprehensive overview of scientific production between 2018 and 2023. Based on the findings, the annual growth rate of 6.72% indicates significant dynamicity in academic production, while the average of 12.14 citations per paper reveals the impact and relevance in the scientific community. Collaborations among authors were also evident, with 25.71% consisting of international collaborations. This underscores the global nature of the research. As for the evaluation by Lotka's law, it confirmed an uneven distribution in paper production, with the majority written by a single author. Meanwhile, Bradford's law revealed the concentration of activity in a small number of leading journals. Finally, mapping the collaborations across countries revealed interesting patterns, with China emerging as a central player and the United States engaging in numerous collaborations. These findings underscore the importance of international cooperation in scientific research. Overall, this study provides a comprehensive and nuanced view of scientific production in the sample period.

## Figures and Tables

**Table 1:** Search strategy.

**Number**	**Thesaurus**
#1	(“visible light” OR “visible radiation” OR “visible spectrum” OR “blue light” OR “yellow light” OR “red light” OR “light spectrum” OR “daylight” OR “electromagnetic radiation” OR “illumination” OR “radiant energy” OR “visible radiation” OR “electromagnetic spectrum”)
#2	(“skin photoaging” OR “skin photodamage” OR “photoaged skin” OR “photodamaged skin” OR “premature skin aging” OR “UV-induced skin aging” OR “skin aging” OR “photodamage” OR “premature aging of the skin” OR “cutaneous aging” OR “photoaging” OR “UV-induced skin damage” OR “premature skin aging” OR “UV-induced damage”)
#3	PUBYEAR >2017 AND PUBYEAR <2024
#1 AND #2 AND #3	

**Table 2:** Main data information.

**Characteristics**	**Results**
Time	2018:2023
Sources	180
Documents	280
Annual growth rate (%)	6.72
Document average	3.25
Average citations	12.14
References	15271
Keywords plus	3234
Author's keywords	788
Authors	1346
Authors of single-authored docs	14
Single-authored	15
Coauthors per document	5.6
International coauthorships (%)	25.71
Article	205
Book chapter	13
Conference paper	7
Conference review	1
Review	51
Short survey	3

**Table 3:** Top 10 scientific referees.

**Institution**	**Country/region**	**Scholarly output**	**View count**	**Field-weighted citation impact**	**Citation count**
The National Centre for Scientific Research	France	15	399	1.64	275
Chinese Academy of Sciences	China	11	171	1.13	160
Henry Ford Health System	United States	11	345	2.47	191
Institut National de la Santé et de la Recherche Médicale	France	9	216	1.84	158
Fudan University	China	7	134	1.24	29
Universidade de São Paulo	Brazil	7	340	1.89	142
University of Alcalá	Spain	7	124	3.19	233
Commissariat à l'énergie Atomique et aux Énergies Alternatives	France	6	208	1.8	136
Leibniz Research Institute for Environmental Medicine	Germany	6	240	2.82	189
Russian Academy of Sciences	Russia	6	153	0.69	67

**Table 4:** Top 10 most productive scientific journals.

**Scopus source**	**Scholarly output**	**View count**	**Field-weighted citation impact**	**Citation count**
International Journal of Molecular Sciences	8	187	1.18	141
Journal of Cosmetic Dermatology	8	253	1.79	198
Photodermatology, Photoimmunology and Photomedicine	7	201	2.05	98
Photosynthesis Research	6	123	0.7	42
Scientific Reports	6	158	0.5	107
Biomedical Optics Express	5	87	0.79	37
ACS Applied Materials & Interfaces	4	102	1.46	98
Frontiers in Plant Science	4	45	1.39	72
Photochemical and Photobiological Sciences	4	117	0.85	27
Photochemistry and Photobiology	4	103	0.74	95

## Data Availability

The data supporting this study was derived from prior research. The corresponding author can provide the data upon request.
